# Advanced Engineering Technology in Orthopedic Research

**DOI:** 10.3390/bioengineering11090925

**Published:** 2024-09-15

**Authors:** Rongshan Cheng, Huizhi Wang, Cheng-Kung Cheng

**Affiliations:** 1 School of Biomedical Engineering & Med-X Research Institute, Shanghai Jiao Tong University, Shanghai 200030, China; chengrongshan@sjtu.edu.cn; 2 Engineering Research Center of Digital Medicine and Clinical Translation, Ministry of Education, Shanghai 200030, China; 3 Department of Orthopaedic Surgery, Shanghai Ninth People’s Hospital, Shanghai Jiao Tong University School of Medicine, Shanghai 200011, China; 4 Center for Intelligent Medical Equipment and Devices, Institute for Innovative Medical Devices, University of Science and Technology of China, Hefei 230026, China; wang_huizhi8866@163.com; 5 Suzhou Institute for Advanced Research, University of Science and Technology of China, Suzhou 215123, China

Musculoskeletal injuries are increasing in conjunction with the aging of populations and the rising frequency of exercise. It is essential to develop engineering technologies to prevent these injuries and improve the current treatment and rehabilitation strategies to facilitate a rapid return to their daily lives.

In recent years, there have been significant developments in implant design, surgical techniques, rehabilitation protocols, and technologies for injury prevention. For example, novel biomechanical technologies for in vitro and in vivo examination, advanced medical imaging techniques, gait analysis, neuromuscular control strategies, and so on. Novel biomechanical technologies have enabled detailed in vitro and in vivo musculoskeletal function analyses, significantly improving diagnostic accuracy, which is crucial for effective treatment [1]. Advanced medical imaging techniques have brought more accurate preoperative planning and treatment assessment [2]. Biocompatible materials, customized implants, and optimized surgical techniques have been demonstrated to improve post-surgery body function [3]. Gait analysis and associated training equipment can be integrated for intelligent rehabilitation and health monitoring [4]. Although these technological advancements have significantly improved patient outcomes, postoperative complications, such as aseptic loosening of implants [5], improper biomechanics during gait [6], long-term degenerative changes in adjacent tissues [7], and graft failures [8], remain common. These complications severely limit patient mobility and may predispose patients to additional surgeries. Therefore, there is still a critical need to improve current surgical techniques and rehabilitation approaches to restore the body’s functions. Moreover, new innovative strategies and smart wearable devices must be developed to prevent related injuries and aid in rehabilitation.

The current Special Issue of *Bioengineering* is dedicated to exploring the latest advanced engineering technologies applied to orthopedic research. This collection presents cutting-edge innovations and methodologies with the potential to transform these fields. Eight papers have been accepted to form this particular issue.

The first article, entitled “Interactive Game-Based Platform System for Assessing and Improving Posture Control in the Elderly” and authored by Pi-Chang Sun et al. [9], investigates the development and implementation of an interactive game-based platform designed to assess and improve postural control in elderly individuals. The platform incorporates a computerized system with a force plate to measure the center of pressure (COP) displacement and provide real-time feedback during balance training exercises. The study highlights the age-related decline in postural control and emphasizes improving this function to reduce fall risks in the elderly. The game-based training program includes multi-directional weight-shifting exercises, during which the participants try to move their COP within their stability limits. These exercises are designed to improve anterior–posterior and medial-lateral balance control, which is critical for maintaining stability during daily activities. The study finds significant balance improvements in the experimental group when compared with the control group, suggesting its potential application in improving postural control and preventing fall risk in elderly individuals.

The second article, “Development and Validation of an Artificial Intelligence Preoperative Planning and Patient-Specific Instrumentation System for Total Knee Arthroplasty”, authored by S. Li et al. [10], focuses on developing and validating an AIJOINT system for preoperative planning and designing patient-specific instrumentation (PSI) for total knee arthroplasty (TKA). The AIJOINT system uses the 3D-UNet for image segmentation and the modified HRNet neural network structures for identifying anatomical landmarks. The 3D-UNet, which includes an encoding section and a decoding section, is a deep learning model designed for precise image segmentation. The modified HRNet starts with high-resolution feature images as the first stage, progressively adding high- to low-resolution feature images and transferring the multiresolution feature images in parallel connections, which minimizes feature loss and improves recognition accuracy. The article reports that the AIJOINT system achieved higher accuracy in predicting the optimized femoral and tibial component sizes than traditional methods, with significant reductions in processing time. In addition, the AIJOINT-designed PSI demonstrates improvements in surgical outcomes, including accuracy of lower limb alignment and reduced blood loss. Overall, this study highlights the potential of the artificial intelligence-based AIJOINT to revolutionize preoperative planning in orthopedic surgery, providing a more efficient and accurate approach to arthroplasty.

The third article, entitled “Biomechanical and Biological Assessment of Polyglycerol Sebacate-Coupled Implant with Shape Memory Effect for Treating Osteoporotic Fractures” and authored by Suzy Park et al. [11], presents the development of an innovative implant combining polyglycerol sebacate (PGS) with a shape memory effect for treating osteoporotic fractures. The research outlines the synthesis process of PGS, which involves curing under specific conditions to improve its mechanical properties and ensure biocompatibility. The PGS is capable of providing shape memory effects and allows for a free form, which can remember the original shape and obtain a temporary shape under melting point and then recover its original shape at body temperature. Because these properties can easily produce customized shapes, the PGS membrane in this study is coupled with a traditional implant, and the resulting hybrid is subjected to various biomechanical tests, including assessments of pull-out strength and maintenance strength. These tests show that the PGS-coupled implants had significantly higher fixation strength than conventional implants. In addition, the study includes in vitro cytocompatibility tests using C2C12 mouse muscular cells, confirming that the PGS membrane supported cell viability and growth. These results suggest that the PGS-coupled implant improves mechanical stability and provides a favorable environment for bone regeneration. Overall, this study highlights the potential of PGS-coupled implants to advance the treatment of osteoporotic fractures.

The fourth article, entitled “Development and Validation of a Novel In Vitro Joint Testing System for Reproduction of In Vivo Dynamic Muscle Force” and authored by Yangyang Yang et al. [12], designs a novel in vitro joint testing system to accurately reproduce in vivo dynamic muscle forces. The system is developed to address the limitations of traditional testing methods, which often rely on simplified loading scenarios that do not fully reproduce the complex dynamics of muscle forces during functional activities. The study details the development of the muscle loading platform, which includes a control system, an executive mechanism, compliant materials, and measuring equipment. The platform is designed to simulate the forces exerted by muscles on joints during movements, providing a more realistic assessment of joint mechanics. The system uses a numerical computation method to reproduce the muscle forces based on dynamic physiological muscle loadings. The authors conduct tests on the knee joint to validate the platform’s performance, focusing on three muscles: the gastrocnemius lateralis, the rectus femoris, and the semitendinosus. The results show that the platform could accurately reproduce the magnitude and changing pattern of muscle forces with minimal error and high robustness. Overall, this study highlights the developed platform has great potential to be applied in a future musculoskeletal loading system.

The fifth article, entitled “Pullout Strength of Pedicle Screws Inserted Using Three Different Techniques: A Biomechanical Study on Polyurethane Foam Block” and authored by Lien-Chen Wu et al. [13], investigates how the inserting technique affects the pullout strength of pedicle screws. Pedicle screws are used in spinal surgeries to provide stability and support. The study focuses on three different pedicle screw designs: single-lead-thread (SLT), dual-lead-thread (DLT), and mixed-single-lead-thread (MSLT) screws. These screws are inserted into rigid polyurethane foam blocks to evaluate their performance under different conditions. The study examines the effects of three insertion strategies, with and without cyclic loading of the screws before pull-out tests: (A) screw inserted to a depth of 33.5 mm, (B) screw inserted to 33.5 mm and then reversed by 3.5 mm to simulate an adjustment of the tulip height of the pedicle screw, and (C) screw inserted to a depth of 30 mm. The results indicate that screws inserted using strategy A consistently demonstrate the highest pullout strength across all screw types with or without cyclic loading, and the MSLT screws showed superior overall performance without cyclic loading. However, the pullout strength of all screws decreases significantly with cyclic loading, particularly for those inserted using strategy B. The MSLT screws experience the most significant reduction in pullout strength under these conditions, highlighting the importance of avoiding screw adjustment after initial insertion to maintain fixation strength. Overall, the study provides valuable insights into the biomechanical performance of different pedicle screw designs and insertion techniques and offers recommendations to optimize surgical outcomes in spinal fixation procedures.

The sixth article, entitled “Larger Medial Contact Area and More Anterior Contact Position in Medial-Pivot than Posterior-Stabilized Total Knee Arthroplasty during In-Vivo Lunge Activity” and authored by Diyang Zou et al. [14], investigates the in vivo kinematics and articular contact characteristics of medial-pivot (MP) TKA and posterior-stabilized (PS) TKA during a weight-bearing lunge activity. The study aims to determine which TKA design better reproduces the natural knee movement patterns and stability. The results show that PS-TKA exhibited significantly more posterior femoral translation than MP-TKA at high flexion angles, which may increase shear forces and wear on the polyethylene insert. In contrast, the MP-TKA has a larger contact area and a more stable medial pivot rotation during high flexion, suggesting improved medial stability. Both TKA designs show similar clinical outcomes one year (MP-TKA) or two years (PS-TKA) following surgery. Still, the in vivo kinematic differences highlight the potential advantages of MP-TKA in providing enhanced medial stability and reducing stress on the implant. Overall, this study provides insights into the biomechanical performance of MP-TKA compared to PS-TKA and suggests that MP-TKA may offer better long-term durability and patient satisfaction due to its superior medial stability during high-flexion activities.

The seventh article, entitled “Gait Analysis to Monitor Fracture Healing of the Lower Leg” and authored by Elke Warmerdam et al. [15], reviews the use of gait analysis to monitor the healing process of lower leg fractures. Traditionally, the progress of fracture healing is typically monitored by radiation exposure from X-rays. The authors investigate whether gait analysis, including spatiotemporal gait parameters, kinematics, kinetics, and pedography, can provide a more timely and non-invasive method for assessing fracture healing. The article discusses how changes in gait patterns can reflect the progress of fracture healing, particularly in spatiotemporal parameters such as gait speed, step length, and asymmetry. These parameters improved the healing process in patients with tibial fractures. The review highlights that pedographic measurements, such as pressure distribution during walking, differ significantly between patients with successful fracture healing and those with non-union. These differences suggest that gait analysis could predict healing problems earlier than radiographs. Overall, the review concludes that gait analysis seems to be a valuable tool for monitoring the healing process and predicting the occurrence of non-union lower leg fractures. 

The eighth article, entitled “Inconsistency in Shoulder Arthrometers for Measuring Glenohumeral Joint Laxity: A Systematic Review” and authored by Eluana Gomes et al. [16], reviews the inconsistency in the use of shoulder arthrometers to measure glenohumeral joint laxity. The study systematically evaluates the effectiveness and reliability of different shoulder arthrometers in measuring glenohumeral joint laxity, particularly in healthy individuals, athletes, and those with shoulder injuries. The article also discusses the significant variation in laxity measurements between different shoulder arthrometers and testing conditions, including the type of device, applied load, and patient positioning. The results show that the inconsistency of shoulder arthrometers and the lack of standardization in measurement protocols can lead to unreliable results, which may affect clinical decision-making regarding diagnosing and treating shoulder instability. Overall, the review highlights the need for standardized testing protocols and shoulder arthrometers to ensure accurate and reproducible measurements of glenohumeral joint laxity. 

The papers in this Special Issue guide the readers to the latest advances in advanced engineering technology in orthopedic research. These advances may help improve the effective prevention of musculoskeletal injuries or diseases, enhance the compatibility of implants with the human body, optimize the post-operative biomechanical function of the body, increase the effectiveness and efficiency of post-surgical functional assessments, and provide innovative integrated diagnostic and therapeutic technologies for preventing age-related degenerative diseases and accelerating post-operative recovery. Furthermore, research on new surgical technologies and smart wearable detection techniques should be continuously advanced to further reduce post-operative complications and improve the body function and daily activities of patients with musculoskeletal diseases.
